# Inhibition of the Sp1/CTR1 axis attenuates cuproptosis and alleviates axonal injury following intracerebral hemorrhage

**DOI:** 10.3389/fmolb.2026.1932767

**Published:** 2026-09-14

**Authors:** Ke Zhang, Wanyu Ma, Likun Wang, Guofeng Wu, Anjun Song, Siying Ren

**Affiliations:** 1 Emergency Department, The Affiliated Hospital of Guizhou Medical University, Guiyang, Guizhou, China; 2 Key Lab of Acute Brain Injury and Function Repair in Guizhou Medical University, Guiyang, Guizhou, China

**Keywords:** Ctr1, cuproptosis, intracerebral hemorrhage, neuronal axon, Sp1

## Abstract

Intracerebral hemorrhage (ICH) causes severe neurological deficits, and secondary neuronal injury is a major barrier to recovery. Cuproptosis, a copper-dependent form of regulated cell death, has been linked to metabolic stress, but its role in axonal injury after ICH remains unclear. Here, we used *in vivo* rat ICH models and *in vitro* ICH-like neuronal injury models to examine changes in copper homeostasis, cuproptosis-associated molecular alterations, and axonal injury following ICH. Copper levels were markedly increased in perihematomal brain tissue and injured neurons, accompanied by reduced mitochondrial protein lipoylation and increased dihydrolipoamide S-acetyltransferase oligomerization, consistent with cuproptosis-associated molecular abnormalities. Specificity protein 1 (Sp1) and the copper transporter 1 (CTR1) were also significantly upregulated and showed similar temporal patterns. In parallel, ICH induced axonal injury-related molecular changes, including increased amyloid precursor protein and decreased growth-associated protein 43 expression. Pharmacological inhibition of Sp1 with plicamycin reduced CTR1 expression, attenuated copper overload, ameliorated cuproptosis-associated molecular abnormalities, and improved the measured axonal injury-related molecular and morphological changes *in vivo* and *in vitro*. These findings suggest that Sp1/CTR1-associated copper uptake regulation may contribute to secondary neuronal injury after ICH and may represent a potential therapeutic entry point for further investigation.

## 1 Introduction

Intracerebral hemorrhage (ICH) is the stroke subtype with the highest rates of disability and mortality, and survivors often suffer from severe and persistent neurological deficits (Puy et al., 2023). In addition to primary injury caused by vascular rupture and hematoma-induced compression of surrounding brain tissue, secondary injuries, such as inflammatory responses, blood–brain barrier disruption, oxidative stress, and cell death, are critical determinants of clinical outcome (Shao et al., 2019; Alsbrook et al., 2023). Although various therapeutic strategies targeting these pathological processes have been proposed, their clinical benefits remain limited (Shoamanesh and Katsanos, 2021; Chiang et al., 2025), indicating that key molecular mechanisms underlying post-ICH injury have yet to be fully elucidated.

Neuronal death represents an important pathological event in secondary brain injury following ICH. Hematoma degradation products can rapidly trigger inflammatory cascades and induce oxidative stress and metabolic disturbances, thereby exacerbating neuronal injury (Bai et al., 2020). Although multiple forms of programmed cell death, including apoptosis, necroptosis, and ferroptosis, have been implicated in post-ICH neuronal damage, interventions targeting these pathways have not led to significant improvements in neurological outcomes (Song et al., 2022; Zhang et al., 2022; Mao et al., 2025). These observations suggest the existence of additional, insufficiently characterized cell death mechanisms.

Cuproptosis is a recently proposed form of copper-dependent programmed cell death, characterized by the direct binding of copper ions to mitochondrial lipoylated proteins, leading to their abnormal aggregation, subsequent proteotoxic stress, and ultimately cell death (Tsvetkov et al., 2022). During this process, mitochondrial metabolic factors regulate cellular susceptibility to cuproptosis by modulating lipoylation status and copper-dependent metabolic activity. Among them, ferredoxin 1 (FDX1) and lipoic acid synthetase (LIAS) are considered positive regulators of cuproptosis, promoting copper-dependent toxicity by enhancing copper reduction activity and providing substrates for lipoylation, respectively. In contrast, glutaminase (GLS) negatively regulates cuproptosis by modulating the metabolic flux of glutamine into the tricarboxylic acid (TCA) cycle and thereby influencing the copper-dependent metabolic state of mitochondria. Meanwhile, abnormal changes in lipoylated proteins such as dihydrolipoamide S-acetyltransferase (DLAT), together with their oligomerization, are regarded as important molecular features of cuproptosis (Solmonson and DeBerardinis, 2018; Tsvetkov et al., 2019; Martins et al., 2020).

Copper homeostasis is essential for normal central nervous system function. As a cofactor for numerous enzymes, copper participates in redox reactions, neurotransmitter synthesis, and synaptic plasticity, and contributes to antioxidant defense through pathways involving copper–zinc superoxide dismutase (Cu/Zn-SOD) (Robinett et al., 2018; Gale and Aizenman, 2024). However, copper is a double-edged sword: while physiological levels are indispensable for cellular survival and function, disruption of copper homeostasis—whether due to deficiency or pathological accumulation—can result in cellular dysfunction and cytotoxicity. After ICH, pathological processes such as blood–brain barrier disruption and hematoma degradation lead to abnormal distribution of metal ions within the injured region, creating conditions for intracellular copper dyshomeostasis (Madangarli et al., 2019; Pushie et al., 2024). Abnormal copper homeostasis is closely associated with dysregulated cellular copper uptake, suggesting that copper transport pathways may play an important role in post-ICH copper imbalance.

Copper transporter 1 (CTR1) is the principal transporter responsible for cellular copper uptake, and its expression level is an important determinant of intracellular copper load (Kim et al., 2013). Overexpression of CTR1 markedly increases cellular sensitivity to copper and induces copper-dependent cell death (Luo et al., 2025). At the transcriptional level, specificity protein 1 (Sp1) binds to the promoter region of CTR1 and promotes its transcription, thereby contributing to the regulation of cellular copper uptake and copper homeostasis (Liang et al., 2012). Inhibition of Sp1 activity has been reported to downregulate CTR1 expression and attenuate cuproptosis-related injury (Yong et al., 2023; Chen et al., 2024). However, whether Sp1/CTR1-mediated copper dyshomeostasis occurs in neurons after ICH, and whether this pathway contributes to cuproptosis-associated secondary neuronal and axonal injury, remain poorly understood.

Neuronal axonal injury and alterations in axonal integrity–related markers are key factors limiting neurological recovery after ICH, particularly when the basal ganglia and internal capsule are involved (Hijioka et al., 2016). Such injury not only directly disrupts neural circuits, but neuronal death further induces axonal degeneration and transport dysfunction, thereby exacerbating circuit damage (Katsuki and Hijioka, 2017). Against this background, a critical scientific question arises: is Sp1/CTR1 axis–mediated copper dyshomeostasis after ICH associated with cuproptosis-associated molecular alterations and axonal injury-related changes, thereby contributing to secondary neurological impairment?

Focusing on cuproptosis, the present study investigates its role in secondary neural injury following ICH, with particular emphasis on copper homeostasis regulation mediated by the Sp1/CTR1 axis. By examining its association with neuronal injury and axonal injury–related changes, this work aims to deepen the understanding of post-ICH secondary brain injury from the perspective of metabolic homeostasis.

## 2 Materials and methods

### 2.1 Materials

#### 2.1.1 Main instruments

A stereotaxic apparatus for Sprague–Dawley (SD) rats (ZH-Lanxing Type B, Zhenghua Biological Instrument Equipment Co., Ltd., China) was used. Protein analysis experiments were performed using gel electrophoresis and transfer systems (Bio-Rad, Hercules, CA, United States) and a chemiluminescence imaging system (Tanon, Shanghai, China). Gene expression analysis was conducted using a real-time quantitative polymerase chain reaction (real-time PCR) system (SLAN-96P Real-Time PCR System, Shanghai Hongshi Medical Technology Co., Ltd., Shanghai, China). Immunofluorescence imaging was carried out with a laser scanning confocal microscope (Olympus, Tokyo, Japan). Cell culture–related experiments were performed in a carbon dioxide incubator (Thermo Fisher Scientific, Waltham, MA, United States).

#### 2.1.2 Main reagents

Plicamycin (also known as Mithramycin A, MA, MedChemExpress, United States, HY-A0122), a classical Sp1 DNA-binding inhibitor, was used as a pharmacological tool to inhibit Sp1 DNA-binding activity in this study. The immortalized rat cortical neuronal cell line RN-C was obtained from Qingqi Biotechnology Development Co., Ltd. (China; Cat. No. BNF608006418), and primary rat cortical neurons were purchased from Procell Life Science & Technology Co., Ltd. (China; Cat. No. CP-R105). Primary antibodies for Western blotting were mainly purchased from Proteintech (United States), including Sp1 (21962-1-AP), LIAS (11577-1-AP), FDX1 (12592-1-AP), GLS (12855-1-AP), DLAT (13426-1-AP), APP (60342-1-Ig), and GAP43 (16971-1-AP). Additional antibodies included CTR1 (Bioswamp, China, PAB36840), Lipoic Acid (Santa Cruz Biotechnology, United States, sc-101354), and glyceraldehyde-3-phosphate dehydrogenase (GAPDH, Servicebio, China, ZB15004-HRP). RNA extraction kit (Yeasen, China, 19221ES50), reverse transcription kit (Yeasen, China, 11151ES10), and SYBR Green real-time PCR kit (Yeasen, China, 11184ES08) were used. ChIP experiments were performed using a ChIP kit (Millipore, United States, 17–295), together with anti-Sp1 antibody (Thermo Fisher Scientific, United States, MA5-35331), anti-Histone H3 antibody (Abcam, United Kingdom, ab1791), and IgG (Abcam, United Kingdom, ab172730). For immunofluorescence experiments, Proteintech antibodies (United States) included Sp1 (66508-1-Ig), APP (60342-1-Ig), and voltage-dependent anion channel 1 (VDAC1, 10866-1-AP), while Bioss antibodies (China) included CTR1 (bs-10773R) and neuronal nuclei (NeuN, bs-10394R). Detailed information on the antibodies used in this study is provided in Supplementary Table S1.

### 2.2 Methods

#### 2.2.1 Experimental animals

All animal experiments were conducted using specific pathogen-free (SPF) male Sprague–Dawley (SD) rats, approximately 8 weeks of age and weighing 250–300 g. Animals were purchased from Henan Skbex Biotechnology Co., Ltd. (China; license No. SCXK (Yu) 2025-0005). All animal experimental protocols were approved by the Animal Ethics Committee of Guizhou Medical University (approval No. 2403622) and were performed in accordance with relevant guidelines and regulations. This study is reported in accordance with the ARRIVE guidelines. Rats were housed under controlled conditions at 23 °C and approximately 40% relative humidity, with a standard 12 h light/dark cycle and free access to food and water. Animals were acclimatized before experimentation and randomly assigned to different experimental groups. Following behavioral assessment, brain tissues collected from the same animals were used for Western blotting and RT-qPCR, whereas independent cohorts were used for ICP-MS and transcriptomic analyses. Sample sizes were determined based on the study design and prior experimental experience with the model. The number of animals used in each experiment is indicated in the corresponding figure legends.

#### 2.2.2 Establishment of autologous blood ICH model in rats

Rats were fasted for 12 h before surgery with free access to water. After weighing, anesthesia was induced by intraperitoneal injection of 1% pentobarbital sodium (40 mg/kg) (Li et al., 2017). Upon confirmation of loss of pain reflexes, animals were placed in the prone position and secured in a stereotaxic apparatus, with the bregma and lambda positioned at the same horizontal plane. Following hair removal and routine disinfection, a midline scalp incision was made to expose the skull. According to the rat brain stereotaxic atlas, a burr hole was drilled at 0.3 mm anterior to the bregma and 3.0 mm lateral to the right of the midline. Subsequently, 110–120 μL of autologous whole blood was collected from the tail artery. A microsyringe was vertically mounted on the stereotaxic frame, and the needle was slowly advanced to a depth of 6.0 mm into the basal ganglia. A total of 100 μL of autologous blood was then injected evenly over 2 min. In the Sham group, an equal volume of sterile saline was injected instead of autologous blood. After injection, the needle was left in place for 20 min before being slowly withdrawn. The burr hole was sealed with bone wax, and the incision was closed in layers. Rats were allowed to recover in a temperature-controlled environment and then returned to their home cages. To prevent postoperative infection, penicillin was administered intraperitoneally after model induction (50,000 U/kg/day) for three consecutive days.

#### 2.2.3 *In vivo* drug treatment

Plicamycin used in the animal experiments was freshly prepared in 0.9% sterile saline immediately before use. During preparation, the working solution was thoroughly mixed by vortexing and briefly sonicated to ensure complete dissolution and clarity without visible precipitates. Immediately after ICH induction, rats in the ICH + MA group received a single intraperitoneal injection of plicamycin at 0.4 mg/kg (Zou et al., 2024). Animals were euthanized 72 h after ICH induction for sample collection.

#### 2.2.4 Neurological function assessment

Neurological deficits were assessed using the modified Garcia score. This scoring system provides a comprehensive evaluation of neurological function in rats, encompassing parameters such as spontaneous activity, postural symmetry, and forelimb movement. All behavioral assessments were conducted at the designated time points according to the experimental design and were independently performed by two observers who were blinded to the experimental grouping, in order to minimize subjective bias.

#### 2.2.5 Cell culture and *in vitro* ICH-like injury model

The immortalized rat cortical neuronal cell line RN-C and primary rat cortical neurons were cultured under the conditions recommended by their respective suppliers in a humidified incubator at 37 °C with 5% CO_2_. An *in vitro* ICH-like injury model was established by treatment with hemin at a final concentration of 30 μM, according to commonly used protocols reported in previous studies (An et al., 2022). Primary neurons were used exclusively for axon-related immunofluorescence staining and morphological analyses, whereas all other *in vitro* experiments were performed using the immortalized cell line.

#### 2.2.6 Brain tissue preparation

Rats were deeply anesthetized with 1% pentobarbital sodium (50 mg/kg, i.p.) and transcardially perfused with normal saline, after which the brains were rapidly removed. For morphological observation, brains were fixed in 4% paraformaldehyde for 24 h, washed with PBS, and coronally sectioned through the needle tract and hematoma center for gross brain and coronal section imaging.

For molecular and biochemical analyses, fresh brains were rapidly placed on ice, and coronal tissue blocks containing the needle tract and hematoma center were prepared according to the same anatomical landmarks across animals. Perihematomal tissue was collected from the ipsilateral basal ganglia within approximately 1–2 mm from the edge of the hematoma, centered on the needle tract, while avoiding the hematoma core and visible blood clot (Lu et al., 2015). For control animals, the corresponding region of the right basal ganglia was collected using the same stereotaxic coordinates and anatomical range. All tissue sampling was performed using a consistent procedure to improve regional comparability across animals. Tissue samples used for ICP-MS, Western blotting, and RT-qPCR were immediately frozen and stored at −80 °C until analysis, whereas samples used for transcriptomic sequencing were preserved in RNAlater solution after collection.

#### 2.2.7 Measurement of copper content in brain tissue and cell samples by ICP-MS

Copper content in perihematomal brain tissue and cell samples was measured using inductively coupled plasma mass spectrometry (ICP-MS). Samples were digested with nitric acid, brought to a defined final volume, and filtered, after which the measurements were performed by Shanghai Yanlumaman Technology Co., Ltd. Blank samples and standard solutions were included for calibration and quality control during analysis. The obtained copper concentrations were normalized to the protein concentration of each sample and used for subsequent statistical analyses.

#### 2.2.8 Transcriptome analysis

Total RNA was extracted from RNAlater-preserved brain tissues of each experimental group using TRIzol reagent. RNA concentration and integrity were assessed, and only samples with an RNA integrity number (RIN) > 7.0 were used for subsequent analyses. Qualified samples underwent mRNA enrichment for the construction of strand-specific RNA sequencing (RNA-seq) libraries, which were then sequenced using a paired-end strategy on an Illumina NovaSeq 6000 platform (LC-Bio Technology Co., Ltd.) generating at least 6 Gb of raw data per sample. Raw reads were filtered using Cutadapt v1.9, and the Q20, Q30, and GC content of clean reads were assessed using FastQC v0.11.9. Clean reads were aligned to the *Rattus norvegicus* GRCr8 reference genome (Ensembl release 112) using HISAT2 v2.2.1. Transcript assembly and expression quantification were performed using StringTie v2.1.6. FPKM values were used for expression visualization, whereas gene-level count data were used for differential expression analysis. Sample consistency was assessed using Pearson correlation analysis and principal component analysis. Differentially expressed genes (DEGs) were identified using DESeq2, with an exploratory screening threshold of *P* < 0.05 and |log2 fold change (FC)| ≥ 0.263 (corresponding to an absolute fold change ≥1.2) (Yang et al., 2025). Functional annotation and pathway enrichment analyses of DEGs were performed using Gene Ontology (GO) and Kyoto Encyclopedia of Genes and Genomes (KEGG) analyses.

#### 2.2.9 Western blot assay

Total protein was extracted from perihematomal brain tissues or cell samples using RIPA lysis buffer (Beyotime, P0013). Samples were lysed on ice and centrifuged at 12,000 × g for 10 min at 4 °C, and the supernatants were collected for protein quantification. Equal amounts of protein were separated by SDS-polyacrylamide gel electrophoresis (SDS-PAGE) and transferred onto polyvinylidene difluoride (PVDF) membranes. After blocking with 5% nonfat milk in TBST for 1 h at room temperature, membranes were incubated with the corresponding primary antibodies at 4 °C overnight. After washing with TBST, membranes were incubated with the appropriate HRP-conjugated secondary antibodies for 1 h at room temperature. GAPDH was detected using a GAPDH-HRP antibody. Protein signals were detected by chemiluminescence and quantified by densitometric analysis using ImageJ software.

DLAT oligomerization was detected using non-denaturing Western blotting. Proteins were extracted under non-denaturing lysis conditions, and samples were prepared using non-denaturing and non-reducing loading buffer (Beyotime, P0016N) without heat denaturation before loading. Electrophoresis was performed using SDS-free native polyacrylamide gels and non-denaturing PAGE running buffer to preserve protein conformation and oligomeric structures as much as possible. The remaining procedures, including transfer, blocking, antibody incubation, and signal detection were performed as described for conventional Western blotting. DLAT oligomerization was assessed qualitatively based on the high-molecular-weight oligomeric signal.

#### 2.2.10 RT-qPCR

Real-time quantitative PCR was performed using a commercial RT-qPCR reagents system from Yeasen Biotechnology Co., Ltd. Total RNA was extracted from brain tissues using an RNA extraction kit, and equal amounts of RNA were reverse-transcribed into complementary DNA (cDNA). Quantitative PCR was then conducted using the SYBR Green method. The expression levels of target genes were normalized to the internal reference gene GAPDH, and relative expression was calculated using the 2^−ΔΔCt method. Primer sequences (5′–3′) are listed in Table 1.

**TABLE 1:** Sequences for primers.

Gene name	Primer direction	Primer sequences (5′–3′)
Sp1	Forward	GGTGGTGGTGGCAATAACGG
	Reverse	CCCTGGGAGTTGTTGCTGTT
CTR1	Forward	GTCCAGACCTGGTTAGGGAT
	Reverse	GAGTCAGCGCAAAGGTCTTC
GAPDH	Forward	GACATGCCGCCTGGAGAAAC
	Reverse	AGCCCAGGATGCCCTTTAGT

#### 2.2.11 Cell viability assay

Cell viability was assessed using the Cell Counting Kit-8 (CCK-8; Vazyme, China; Cat. No. A311-01). RN-C cells were seeded into 96-well plates at a density of 5 × 10^3^ cells per well in 100 μL of complete culture medium and allowed to adhere under standard culture conditions. To determine the appropriate duration for establishing the *in vitro* ICH-like injury model, cells were treated with hemin for the indicated time points, with untreated cells serving as the control. After treatment, 10 μL of CCK-8 solution was added to each well, followed by incubation at 37 °C for 45–60 min. Absorbance was measured at 450 nm using a microplate reader. Blank wells containing medium and CCK-8 reagent without cells were included for background subtraction. Cell viability was normalized to the untreated control group using the following formula: Normalized viability = (OD_treated − OD_blank)/(OD_control − OD_blank).

To determine the working concentration of plicamycin (MA) for subsequent experiments, CCK-8 assays were further performed under both normal and ICH-like injury conditions. Cells in the normal background were treated with different concentrations of MA, whereas cells in the model background were first treated with hemin, immediately followed by indicated concentrations of MA. MA was dissolved in DMSO and diluted with culture medium before use; the final concentration of DMSO was kept consistent among groups and did not exceed 0.1%. Because basal viability differed between normal and ICH-like injury conditions, normalization was performed separately within each background condition. The corresponding 0 nM MA group was used as the reference and set to 1, namely, the Ctrl + DMSO group for normal cells and the Hemin + DMSO group for model cells. Normalized viability was calculated as follows: Normalized viability = (OD_treated − OD_blank)/(OD_0 nM − OD_blank).

Each independent CCK-8 experiment included 3–6 technical wells per condition depending on the assay design, and all experiments were independently repeated at least three times. Statistical analysis was based on independent experiments rather than technical wells.

#### 2.2.12 *In vitro* drug treatment

In the *in vitro* experiments, cells were treated to establish the corresponding models and then subjected to plicamycin intervention. Plicamycin (MA) was first dissolved in dimethyl sulfoxide (DMSO) to prepare a stock solution and subsequently diluted with culture medium to the desired working concentrations. Based on the results of the CCK-8 dose–response experiments performed under both normal and hemin-induced injury conditions, 100 nM was selected as the working concentration for subsequent experiments, as it did not markedly affect cell viability under normal conditions, while relative viability remained approximately 80% under hemin-treated conditions. The duration of drug treatment was determined according to the specific experimental objectives. Cells in the vehicle control group were treated with an equal volume of culture medium containing DMSO, with the final DMSO concentration not exceeding 0.1%. To exclude potential effects of the solvent on experimental outcomes, a vehicle control was included for validation; as no significant effects were observed, all subsequent *in vitro* experiments were conducted using a three-group design (Ctrl, Hemin and Hemin + MA).

#### 2.2.13 ChIP-qPCR

Chromatin immunoprecipitation (ChIP) assays were performed using a ChIP assay kit (Millipore, 17–295) according to the manufacturer’s instructions. Cells were cross-linked with formaldehyde, and chromatin was subsequently fragmented. Immunoprecipitation was carried out using an anti-Sp1 antibody (5.0 μg per reaction, Thermo Fisher Scientific, MA5-35331) to enrich Sp1-bound chromatin. An anti-Histone H3 antibody (5.0 μg per reaction, Abcam, ab1791) was used as a positive control, and rabbit IgG (5.0 μg per reaction, Abcam, ab172730) served as a negative control. After purification of the immunocomplexes, DNA was recovered. Enriched DNA was analyzed by real-time quantitative PCR, and results were expressed as a percentage of input DNA (% input). ChIP-qPCR was performed using n = 3 independent biological replicates per group, with each qPCR assay performed in technical triplicate. ChIP–qPCR primers were designed to target the proximal promoter region of CTR1 (CTR1-F: AAGGCAAATGAGGAGGTGAG; CTR1-R: GTAAAGGCCGCCGATTCT).

#### 2.2.14 Immunofluorescence and probe labeling

Cells were seeded into six-well plates containing sterile coverslips and subjected to the designated treatments once an appropriate density was reached. After treatment, cells were washed with PBS, fixed with 4% paraformaldehyde for 15–20 min at room temperature, and permeabilized with 0.1% Triton X-100 for 10–15 min. After blocking with normal serum from the same host species as the secondary antibody for 30 min, cells were incubated with the corresponding primary antibodies according to the experimental objectives at 4 °C overnight. After three washes with PBS, cells were incubated with matched fluorophore-conjugated secondary antibodies for 1 h at room temperature in the dark. Nuclei were counterstained with DAPI, and coverslips were mounted with antifade mounting medium.

For intracellular copper detection, cells were processed under the same fixation and permeabilization conditions and labeled with a copper ion–specific fluorescent probe (Solarbio, C8720) according to the manufacturer’s instructions. Images of immunofluorescence staining and copper probe labeling were acquired using a laser scanning confocal microscope at the appropriate excitation and emission wavelengths. For the same marker or probe, images from different groups were acquired using identical imaging settings.

Mean fluorescence intensity of CTR1, lipoylation-related signals, APP, and copper probe signals was quantified using ImageJ software after background subtraction. Regions of interest were selected based on DAPI staining and cellular morphology in the merged images. Sp1 subcellular distribution was analyzed using Image-Pro Plus 6.0. Nuclear regions were defined by DAPI staining, and cytoplasmic regions were determined by subtracting the DAPI-positive nuclear area from the whole-cell region. The Sp1 nuclear/cytoplasmic ratio was calculated as the percentage of Sp1-positive signal area in the nucleus divided by that in the cytoplasm.

### 2.3 Statistical analysis

All quantitative data are presented as the mean ± standard deviation. Statistical analyses were performed using GraphPad Prism version 10.1. For comparisons between two groups, two-tailed unpaired Student’s t-test was used. For comparisons among multiple groups, ordinary one-way analysis of variance (ANOVA) followed by Dunnett’s multiple comparisons test was used for prespecified comparisons with the indicated reference group. Adjusted P values were used for post hoc multiple comparisons. A P value <0.05 was considered statistically significant. The exact number of animals or independent biological replicates used for each quantitative assay is indicated in the corresponding figure legends. Technical replicates or repeated measurements, when performed, were used to improve measurement stability and were not treated as independent biological replicates.

## 3 Results

### 3.1 Establishment of the ICH model in SD rats and behavioral assessment

An ICH model was established in SD rats. Across the *in vivo* experiments, a total of 119 rats were used; 115 completed the experimental procedures and were included in the final analyses, while four died following ICH induction before endpoint assessment. Gross specimens and coronal brain sections showed the formation of a stable hematoma in the right basal ganglia of the ICH group, indicating successful model establishment (Figure 1A). Behavioral assessments were performed at different time points (4 h, 12 h, 24 h, 72 h, and 120 h) after ICH using the modified Garcia score. The results showed significant differences in the modified Garcia score among the groups. Compared with the Ctrl groups, the modified Garcia score was reduced after ICH, indicating neurological impairment. The score showed an overall downward trend from 4 h to 72 h, with the lowest mean value observed at 72 h, followed by a partial increase at 120 h (Figure 1B). These results indicate a dynamic progression of neurological deficits after ICH.

**FIGURE 1:**
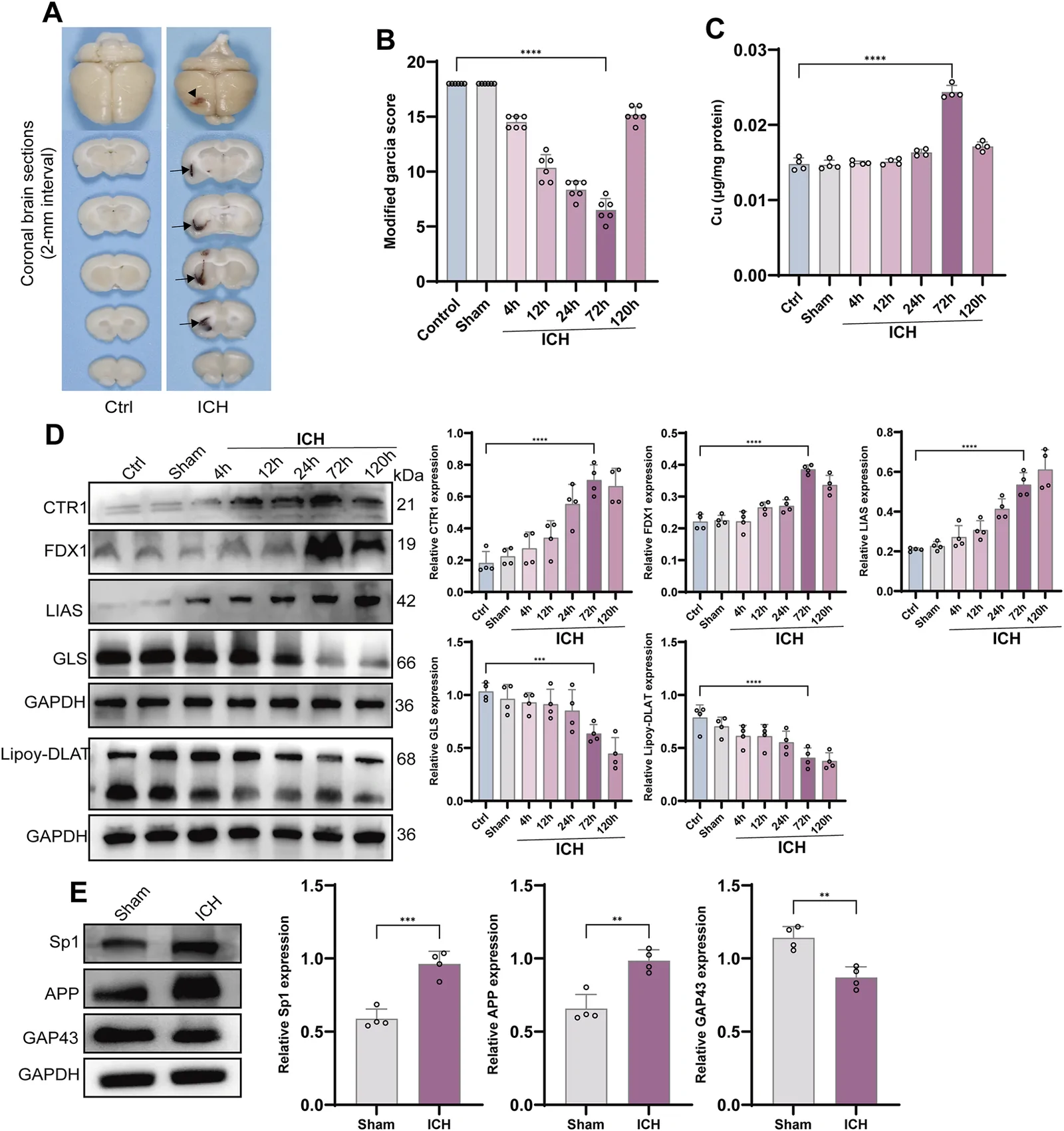
Establishment of the ICH model in SD rats and identification of the optimal observation time point **(A)** Gross brain specimens and coronal brain sections from the normal control (Ctrl) and ICH groups. Triangles indicate the needle entry site, and arrows indicate the hematoma. **(B)** Neurological function of rats at different time points after ICH was evaluated using the modified Garcia score. **(C)** Copper levels in perihematomal brain tissue at different time points after ICH were measured by ICP-MS and normalized to protein concentration. **(D)** Western blot analysis of the copper transporter CTR1 and cuproptosis-associated proteins (FDX1, LIAS, GLS, and lipoylated DLAT) at different time points after ICH; corresponding densitometric quantification is shown on the right. **(E)** Western blot analysis of Sp1 and axonal injury–related proteins (APP and GAP43) in the Sham and ICH groups; corresponding densitometric quantification is shown on the right. Data are presented as mean ± standard deviation. Behavioral assessment was performed with n = 6 animals per group; brain tissue ICP-MS and Western blot analyses were performed with n = 4 independent animal samples per group. Comparisons between two groups were performed using two-tailed unpaired Student’s t-test. Comparisons among multiple groups were performed using one-way ANOVA followed by Dunnett’s multiple comparisons test with the Ctrl group as the reference group. Adjusted P values were used for multiple comparisons. **P < 0.01, ***P < 0.001, ****P < 0.0001. No significant differences were observed between the Ctrl and Sham groups in the parameters assessed where both groups were included (P > 0.05).

### 3.2 Temporal characteristics of perihematomal copper levels and cuproptosis-associated molecular alterations after ICH

Following successful establishment of the ICH model in SD rats, perihematomal brain tissues were collected at different time points (4 h, 12 h, 24 h, 72 h, and 120 h) to evaluate the temporal changes in copper levels and cuproptosis-associated molecular alterations after ICH. ICP-MS analysis revealed that copper levels in perihematomal brain tissue were increased after ICH and peaked at 72 h (Figure 1C). Western blot analysis further demonstrated that the expression of the copper transporter CTR1 and the key cuproptosis-related proteins FDX1 and LIAS showed an overall increasing trend after ICH, whereas the expression of GLS and lipoylated DLAT showed a decreasing trend (Figure 1D). These results indicate that copper accumulation and cuproptosis-associated molecular alterations in perihematomal brain tissue exhibit pronounced time-dependent changes after ICH. Although several changes were already detectable at earlier time points, the 72 h time point showed the most convergent alterations across neurological deficits, copper accumulation, CTR1 expression, and cuproptosis-associated molecular markers. Based on these findings, 72 h was selected as the primary observation time point for subsequent experiments. Thus, the selection of 72 h was data-informed and made after evaluation of the time-course results rather than prespecified.

### 3.3 Upregulation of Sp1 after ICH is accompanied by axonal injury-related changes

On the basis of the observed copper homeostasis imbalance and alterations in cuproptosis-related molecules after ICH, and given that Sp1 has been reported to act as a transcriptional regulator of CTR1 and to participate in copper homeostasis regulation, we further examined changes in Sp1 expression after ICH. Western blot analysis showed that Sp1 protein expression was significantly higher in the ICH group than in the Sham group (Figure 1E). Meanwhile, analysis of axonal injury–related proteins revealed increased APP expression and decreased GAP43 expression in the ICH group compared with the Sham group (Figure 1E). These results show that upregulation of Sp1 after ICH is accompanied by axonal injury–related molecular alterations.

### 3.4 Hemin-induced ICH-Like injury conditions are associated with cuproptosis-associated molecular alterations and axonal injury in neuronal cells

To validate ICH-associated copper homeostasis imbalance and neuronal axonal injury at the cellular level, an *in vitro* ICH-like injury model was established by treating neuronal cells with hemin. Cell viability assays showed a progressive decrease in neuronal viability with prolonged hemin exposure, reaching approximately 50% at 72 h. These data indicate that cellular injury began before 72 h and progressed over time. The 72 h condition was selected because it induced a stable injury phenotype while retaining sufficient viable cells for subsequent molecular and intervention analyses (Figure 2A).

**FIGURE 2:**
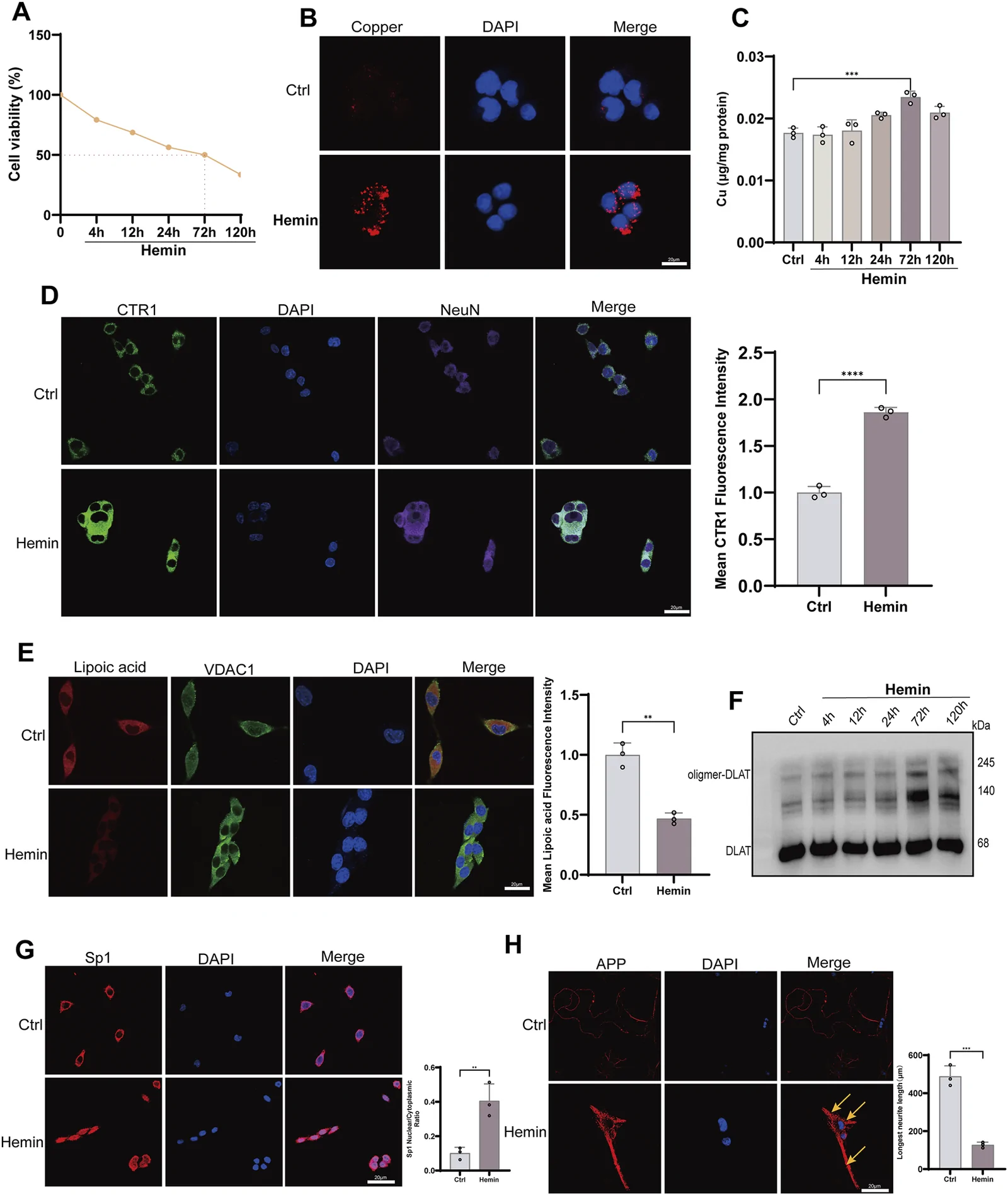
Establishment of the hemin-induced *in vitro* ICH-like injury model and temporal changes in cuproptosis-associated molecular features, Sp1 expression, and axonal injury **(A)** CCK-8 assay showing changes in neuronal cell viability at different time points. **(B)** Intracellular copper accumulation in different groups visualized using a copper ion–specific fluorescent probe. **(C)** Intracellular copper ion concentrations measured by ICP-MS and normalized to protein concentration. **(D)** Immunofluorescence analysis of CTR1 (green) and the neuronal marker NeuN (purple); nuclei were counterstained with DAPI (blue), and corresponding quantitative analysis is shown on the right. **(E)** Immunofluorescence analysis of lipoylation-related signals (red) and the mitochondrial marker VDAC1 (green); nuclei were counterstained with DAPI (blue), and corresponding quantitative analysis is shown on the right. **(F)** Non-denaturing Western blot analysis of DLAT oligomerization following hemin treatment. **(G)** Immunofluorescence analysis of the distribution of the transcription factor Sp1 between the nucleus and cytoplasm, with calculation of the nuclear-to-cytoplasmic ratio to assess Sp1 nuclear translocation. **(H)** Immunofluorescence showing the distribution of the axonal injury–related protein APP (red) and associated axonal morphological alterations; yellow arrows indicate sites of axonal discontinuity, and quantification of the longest axonal length is shown on the right. All immunofluorescence images are representative, scale bar = 20 μm. Data are presented as mean ± standard deviation from n = 3 independent biological replicates per group. **P < 0.01, ***P < 0.001, ****P < 0.0001.

In the hemin-induced neuronal injury model, fluorescence signals from the copper ion–specific probe were markedly enhanced, and quantitative analysis by ICP-MS further confirmed a significant increase in intracellular copper levels (Figures 2B,C). Meanwhile, immunofluorescence staining demonstrated increased CTR1 fluorescence intensity in hemin-treated cells, indicating enhanced neuronal CTR1 expression under ICH-like injury conditions (Figure 2D).

Further analysis of cuproptosis-related molecular features revealed that lipoylation signals were mainly localized to mitochondria and were significantly reduced under ICH-mimicking conditions (Figure 2E). Non-denaturing Western blotting showed an increased high-molecular-weight DLAT oligomeric signal, which was most evident at 72 h (Figure 2F), indicating cuproptosis-associated molecular alterations. These cellular fluorescence findings were in line with the copper accumulation and cuproptosis-associated protein changes observed in perihematomal brain tissue, and were further supported by cellular Western blot analysis (see Supplementary Figure S2).

In addition, immunofluorescence analysis revealed alterations in the nuclear-to-cytoplasmic distribution of Sp1, with an increased nuclear/cytoplasmic ratio under ICH-mimicking conditions, indicating enhanced nuclear translocation of Sp1 (Figure 2G). Regarding axonal morphology, APP immunofluorescence demonstrated impaired axonal structural integrity, accompanied by a significant reduction in the maximum axonal length (Figure 2H).

Collectively, the hemin-treated neuronal model recapitulated several key pathological features observed after ICH at the cellular level, including copper homeostasis imbalance, cuproptosis-related molecular changes, enhanced nuclear translocation of Sp1, and axonal injury, thereby supporting its use for subsequent mechanistic investigations.

### 3.5 Transcriptomic analysis suggests plicamycin-mediated reverse regulation associated with the Sp1/CTR1 axis

To systematically characterize transcriptional remodeling after ICH and to explore the potential targets of plicamycin (MA), transcriptomic sequencing was performed on perihematomal brain tissues from SD rats following ICH. By intersecting genes that were significantly upregulated after ICH with those that were reversely downregulated after MA treatment, a core gene set potentially regulated by MA was identified. Compared with the Sham group, 2665 genes met the screening criteria for upregulation after ICH, of which 779 were downregulated following MA treatment (Figure 3A), indicating that MA partially reverses ICH-induced aberrant gene activation at the transcriptional level.

**FIGURE 3:**
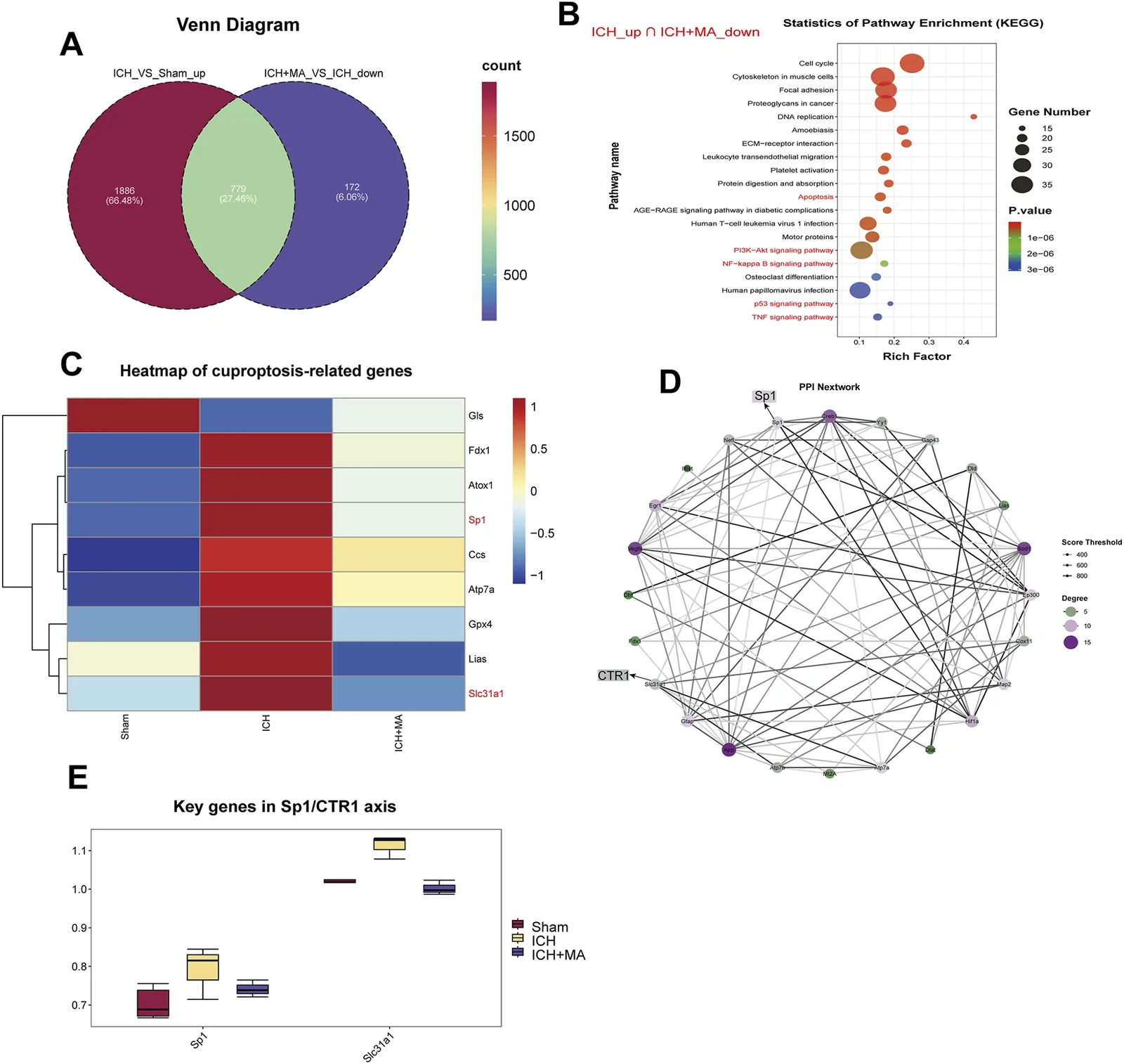
Transcriptomic profiling reveals plicamycin-reversed ICH-induced transcriptional alterations and suggests involvement of the Sp1/CTR1 axis **(A)** Venn diagram showing the intersection between genes upregulated after ICH relative to the control group (ICH vs. Sham, up) and genes downregulated after plicamycin intervention relative to the ICH group (ICH + MA vs. ICH, down), defined as ICH_up ∩ ICH + MA_down. **(B)** KEGG pathway enrichment analysis of the intersecting gene set. **(C)** Heatmap showing the expression patterns of representative cuproptosis-related genes in the Sham, ICH, and ICH + MA groups. **(D)** STRING-based predicted functional association network of the intersecting genes. **(E)** Box plots showing transcript expression levels of Sp1 and Slc31a1 (CTR1) across the indicated groups. Transcriptomic analysis included n = 5, 3, and 3 independent biological samples in the Sham, ICH, and ICH + MA groups, respectively.

KEGG pathway enrichment analysis of the intersecting genes showed predominant enrichment in pathways related to metabolic stress and cell death, including the phosphoinositide 3-kinase–Akt (PI3K–Akt) and tumor protein p53 (p53) pathways (Figure 3B), suggesting that MA intervention may influence multiple regulatory pathways associated with cellular injury after ICH.

Further examination of the cuproptosis-related transcriptional profile revealed that Slc31a1 (CTR1), FDX1, and LIAS were upregulated after ICH and downregulated following MA treatment, whereas GLS was downregulated after ICH and partially restored by MA intervention (Figure 3C). These results indicate that ICH induces transcriptional remodeling associated with copper homeostasis imbalance, which can be partially reversed by MA.

Within the intersecting gene set, the transcription factor Sp1 showed a broadly similar expression pattern to CTR1. The STRING-based network analysis suggested a predicted functional association between Sp1 and CTR1 (Figure 3D). Consistently, expression analysis showed that the mRNA levels of both Sp1 and CTR1 were significantly increased after ICH and synchronously reduced following MA treatment, with a more pronounced reversal observed for CTR1 (Figure 3E). Collectively, transcriptomic analysis suggests that the Sp1/CTR1 axis may be involved in transcriptional regulation associated with copper homeostasis imbalance after ICH and may represent a potential target of MA intervention.

### 3.6 *In Vivo* and in vitro validation of the Sp1/CTR1 axis in the ICH model

Based on transcriptomic analyses showing that Sp1 and CTR1 were synchronously upregulated after ICH and reversely regulated by MA, we further validated the involvement of the Sp1/CTR1 axis in both *in vivo* and *in vitro* models.

At the tissue level, RT–qPCR analysis showed that, compared with the Sham group, the mRNA levels of Sp1 and CTR1 were significantly increased in perihematomal brain tissue of ICH rats, whereas MA intervention markedly reduced the expression of both genes (Figure 4A). Consistently, Western blot analysis demonstrated increased protein expression of Sp1 and CTR1 after ICH, which was significantly attenuated following MA treatment (Figure 4B).

**FIGURE 4:**
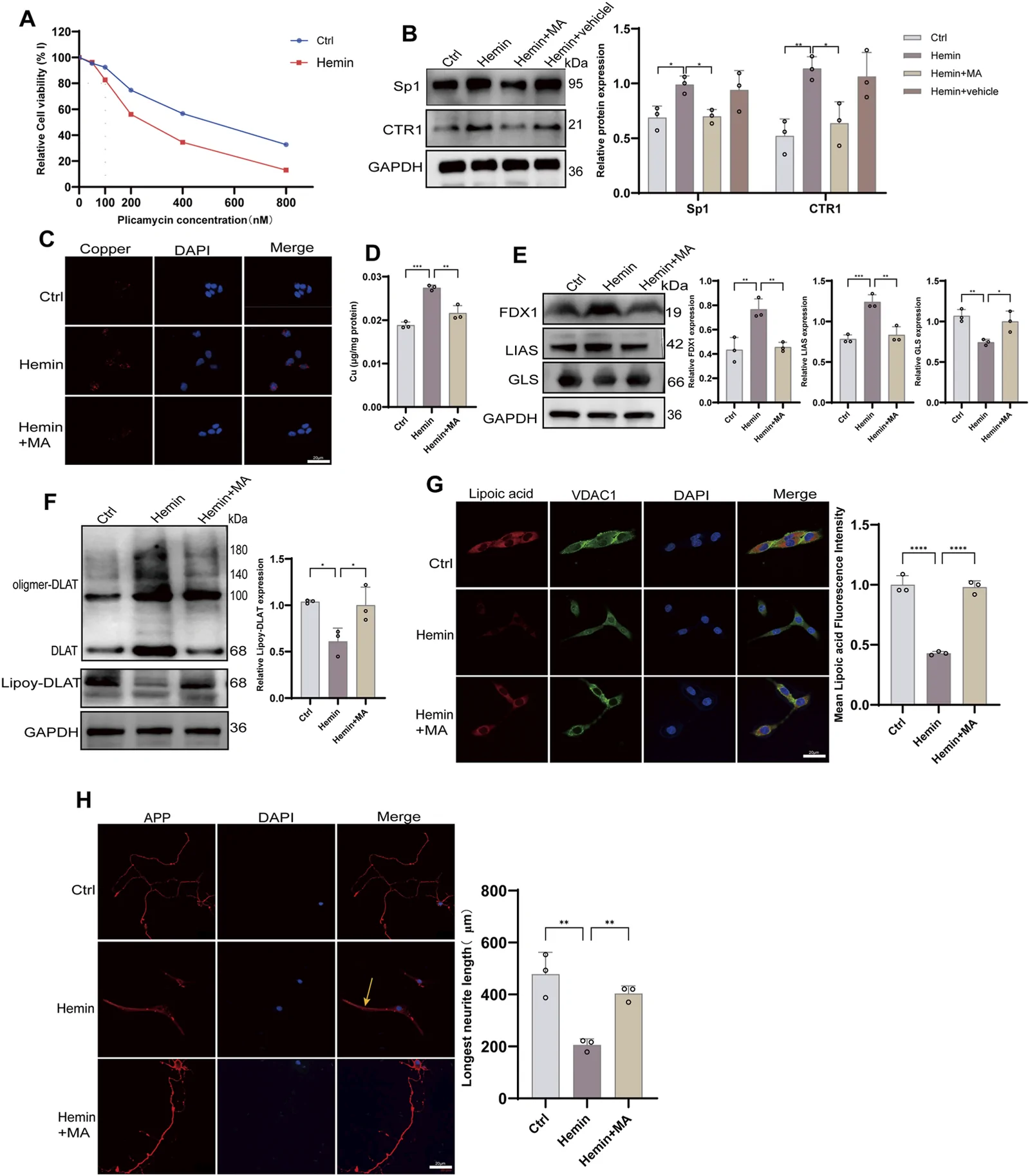
Changes in the Sp1/CTR1 axis in the *in vitro* ICH model and their modulation by plicamycin **(A)** CCK-8 assay showing the effects of different concentrations of plicamycin on cell viability in normal cells and in the *in vitro* ICH-like injury model; data were normalized separately to the corresponding 0 nM MA control within each condition. **(B)** Western blot analysis and densitometric quantification of Sp1 and CTR1 under Ctrl, Hemin, Hemin + MA, and Hemin + vehicle conditions. **(C)** Representative images of intracellular copper accumulation visualized using a copper ion–specific fluorescent probe. **(D)** Quantitative analysis of intracellular copper levels measured by ICP-MS. **(E)** Western blot analysis and densitometric quantification of cuproptosis-associated proteins (FDX1, LIAS, and GLS). **(F)** Western blot analysis of DLAT lipoylation and oligomerization levels; densitometric quantification of lipoylation is shown on the right. **(G)** Immunofluorescence images and quantitative analysis of DLAT lipoylation levels. **(H)** Immunofluorescence staining of APP and quantitative analysis of the longest neurite length; arrows indicate sites of axonal discontinuity. Data are presented as mean ± standard deviation from n = 3 independent biological replicates per group. *P < 0.05, **P < 0.01, ***P < 0.001, ****P < 0.0001.

To minimize interference from tissue heterogeneity, we further performed validation in a hemin-treated RN-C cell model. To exclude potential effects of the solvent, a vehicle control was included, and no significant effect on the measured responses was observed (Figure 4B). Western blot results showed that under ICH-mimicking conditions, Sp1 and CTR1 protein levels were markedly elevated and were significantly reduced after MA treatment (Figure 5C).

Immunofluorescence analysis further revealed enhanced nuclear Sp1 signals under ICH-mimicking conditions, with a significantly increased nuclear-to-cytoplasmic ratio, whereas MA treatment markedly reduced Sp1 nuclear accumulation (Figure 5E). Meanwhile, the mean fluorescence intensity of CTR1 was significantly increased under ICH-mimicking conditions and was substantially decreased following MA intervention (Figure 5F).

Given the role of Sp1 as a transcription factor, we further examined its binding to the CTR1 promoter region using ChIP–qPCR. The results showed that Sp1 enrichment at the CTR1 promoter was significantly increased under ICH-mimicking conditions (Figure 5D).

### 3.7 Modulation of the Sp1/CTR1 axis is associated with attenuation of cuproptosis-associated molecular alterations and axonal injury after ICH

On the basis of the observed activation of the Sp1/CTR1 axis after ICH and its attenuation following MA treatment, we further investigated whether modulation of this axis was associated with attenuation of post-ICH cuproptosis-associated molecular alterations and alleviation of axonal injury-related phenotypes. *In vivo* experiments showed that MA treatment significantly improved neurological function scores in ICH rats and reduced copper levels in perihematomal brain tissue (Figures 6A,B). Meanwhile, MA ameliorated ICH-induced abnormalities in cuproptosis-related molecules, as evidenced by restoration of GLS and lipoylated DLAT levels and marked reductions in the aberrant upregulation of FDX1 and LIAS (Figure 6C). In addition, analysis of axon-related proteins showed that MA treatment decreased APP expression and increased GAP43 expression, an axonal growth–associated marker (Figure 6D).

**FIGURE 5:**
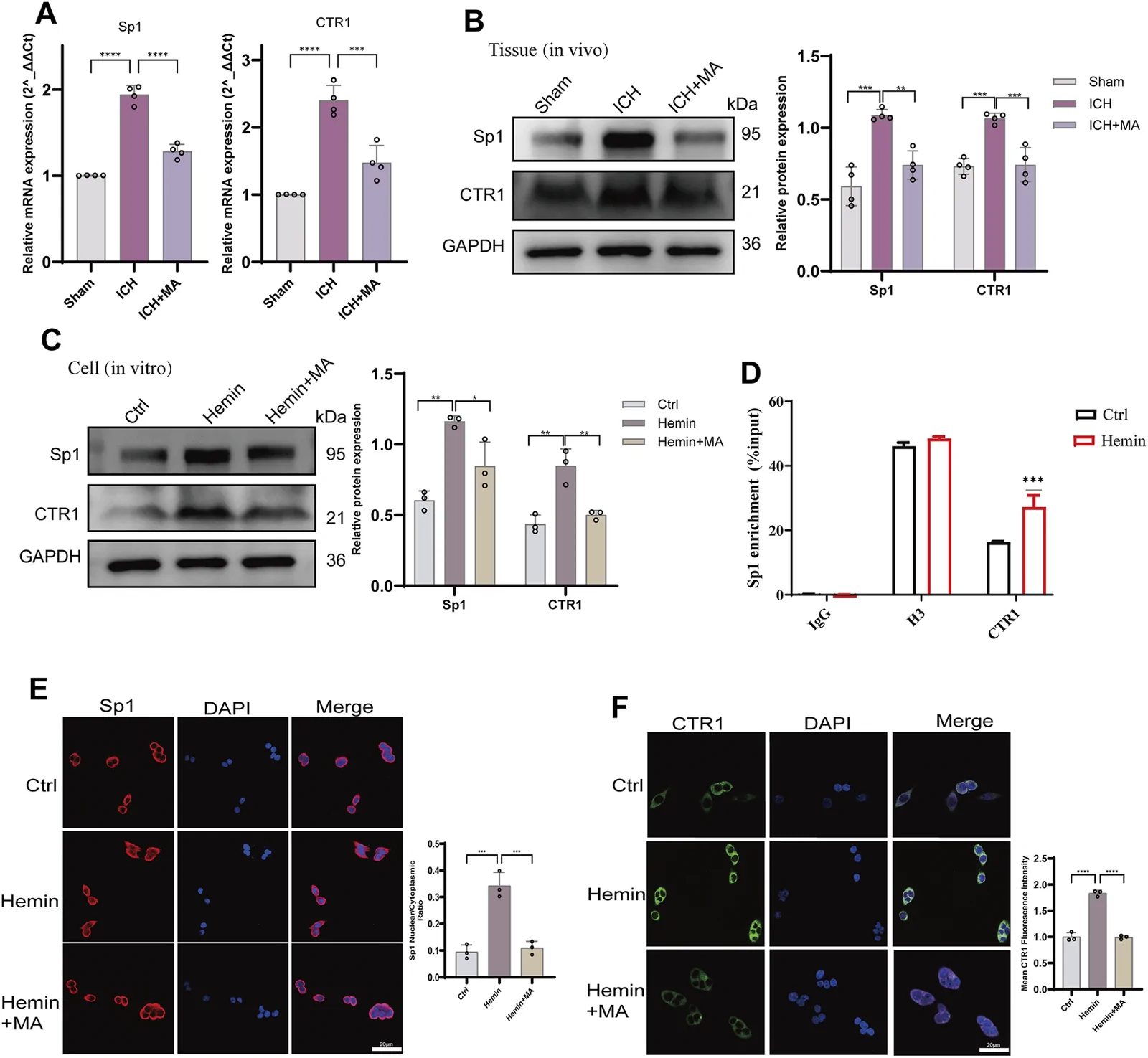
*In vivo* and *in vitro* validation of the Sp1/CTR1 axis **(A,B)** RT-qPCR and Western blot analyses of Sp1 and Slc31a1 (CTR1) mRNA and protein expression in perihematomal brain tissue from SD rats; densitometric quantification of Western blot results is shown on the right. **(C)** Western blot analysis of Sp1 and CTR1 protein expression in the RN-C *in vitro* ICH model; corresponding densitometric quantification is shown on the right. **(D)** ChIP–qPCR analysis of Sp1 enrichment at the Slc31a1 (CTR1) promoter region in hemin-treated RN-C cells. IgG and histone H3 were used as negative and positive controls, respectively, and results were normalized to input DNA and expressed as % input. **(E,F)** Immunofluorescence images of Sp1 and CTR1 with corresponding quantitative analyses. Data are presented as mean ± standard deviation. For *in vivo* experiments in panels A and B, n = 4 independent animal samples per group; for *in vitro* experiments in panels C–F, n = 3 independent biological replicates per group. *P < 0.05, **P < 0.01, ***P < 0.001, ****P < 0.0001.

To validate these findings at the cellular level, we systematically analyzed the effects of MA on the Sp1/CTR1 axis and cuproptosis-related molecular events in a hemin-treated neuronal cell model. CCK-8 assays showed that MA exerted dose-dependent effects on the viability of both normal RN-C cells and hemin-treated cells. At 100 nM, MA did not markedly reduce cell viability under normal conditions, while the relative viability of hemin-treated cells remained approximately 80%; therefore, this concentration was selected for subsequent intervention experiments (Figure 4A). Under this condition, MA significantly suppressed the abnormally elevated expression of Sp1 and CTR1 induced by hemin-induced ICH-like injury, whereas the vehicle control exerted no significant effect on Sp1 or CTR1 expression (Figure 4B).

**FIGURE 6:**
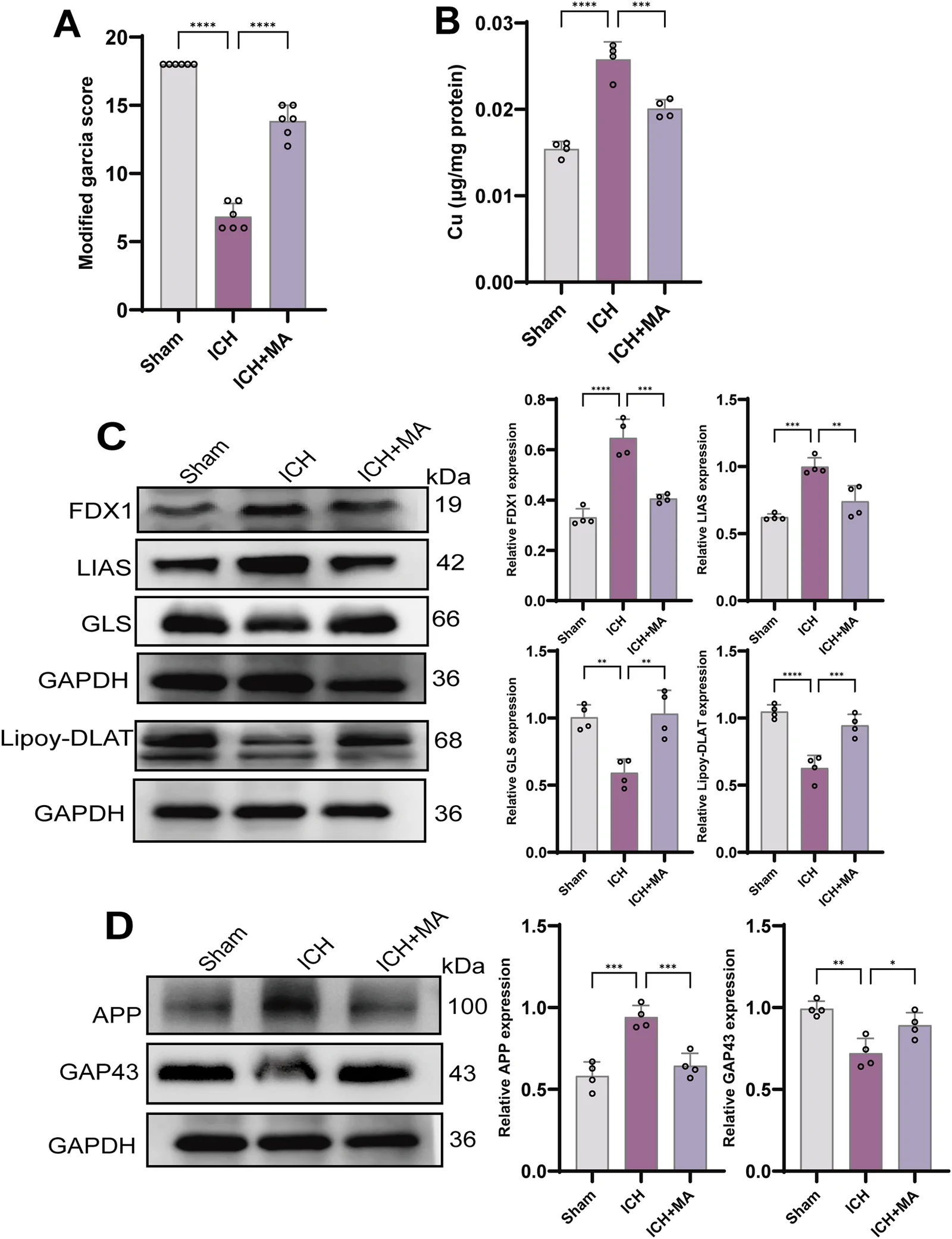
Effects of plicamycin on cuproptosis-associated molecular alterations and axonal injury in rats after ICH **(A)** Neurological function scores of each group assessed using the modified Garcia scoring system. **(B)** Copper levels in perihematomal brain tissue of SD rats measured by ICP-MS. **(C)** Western blot analysis of cuproptosis-related proteins (FDX1, LIAS, GLS, and lipoylated DLAT) in perihematomal brain tissue, with corresponding densitometric quantification shown on the right. **(D)** Western blot analysis of axonal injury-related protein APP and axonal growth–associated protein GAP43, with corresponding densitometric quantification. Data are presented as mean ± standard deviation. Behavioral assessment was performed with n = 6 animals per group; brain tissue ICP-MS and Western blotting were performed with n = 4 independent animal samples per group. *P < 0.05, **P < 0.01, ***P < 0.001, ****P < 0.0001.

Concomitant with downregulation of Sp1/CTR1 expression, intracellular copper fluorescence signals and copper levels were significantly reduced (Figures 4C,D). Further analyses showed that MA treatment attenuated the abnormal upregulation of FDX1 and LIAS under ICH-mimicking conditions and partially restored GLS expression (Figure 4E). Meanwhile, the high-molecular-weight DLAT oligomeric signal was visibly attenuated, and its lipoylation levels exhibited a recovery trend at both the protein and immunofluorescence levels (Figures 4F,G). Immunofluorescence analyses of FDX1, LIAS, and GLS further supported these expression changes (see Supplementary Figure S6).

Along with improvement of copper homeostasis and cuproptosis-related molecular events, axonal injury-related morphological changes were also improved, as reflected by reduced APP-labeled axonal morphological abnormalities and increased neurite length (Figure 4H). Collectively, these results suggest that MA, possibly through inhibition of the Sp1/CTR1 axis, is associated with attenuation of cuproptosis-related changes and improvement of axonal injury-related phenotypes in both *in vivo* and *in vitro* models.

## 4 Discussion

This study investigated the role of copper overload–associated cuproptosis-related changes in neuronal injury following ICH and suggests that the Sp1/CTR1 axis may be involved in this process. Our results show that cuproptosis-associated molecular phenotypes in neurons are markedly enhanced after ICH and closely parallel changes in axonal injury markers, suggesting that these cuproptosis-associated molecular alterations may be associated with the development of post-ICH axonal damage. Furthermore, pharmacological inhibition of Sp1 with MA concurrently attenuated cuproptosis-associated alterations and improved axonal injury-related molecular and morphological changes, indicating that targeting the Sp1/CTR1 axis to modulate cuproptosis-associated molecular injury may provide a novel perspective for understanding secondary neuronal injury after ICH.

In both *in vivo* and *in vitro* models, we consistently observed that the pathological significance of copper homeostasis disruption after ICH extends beyond a mere increase in copper ion levels, with the accompanying cuproptosis-associated molecular alterations representing a potentially important pathological event. Temporal analyses revealed that elevations in copper ion concentration and upregulation of CTR1 did not reach their peak immediately after ICH, but instead progressively intensified with the development of secondary injury, becoming most pronounced at 72 h. This temporal pattern closely aligns with previous reports showing that brain edema peaks at 24–72 h in rat ICH models and that secondary injury rapidly progresses within approximately 72 h after onset in human ICH (Qing et al., 2009; Venkatasubramanian et al., 2011), suggesting that the 72 h time point in our model may represent a stage at which secondary injury–related pathological processes are markedly aggravated. Notably, injury-related changes had already begun before 72 h; therefore, 72 h was selected not as the onset of injury, but as the time point at which cuproptosis-associated molecular alterations were most pronounced under the present modeling conditions. For the *in vivo* experiments, the autologous blood model was selected because it directly exposes perihematomal tissue to whole blood and its components, which better matched our focus on hematoma-related copper dyshomeostasis and secondary neuronal injury. Although the collagenase model can reproduce ongoing bleeding and hematoma expansion, the proteolytic effects of exogenous collagenase may introduce additional tissue injury and complicate mechanistic interpretation. For the *in vitro* experiments, the hemin-treated neuronal model should be interpreted as a simplified ICH-like injury model rather than a complete representation of the perihematomal microenvironment. Hemin is a major hemoglobin degradation product and can induce oxidative and metabolic stress, but it does not incorporate other hematoma-associated factors, such as thrombin, plasma proteins, inflammatory mediators, and mechanical compression. It also does not reproduce the dynamic evolution of the hematoma or the complex interactions among neural, glial, vascular, and infiltrating immune cells. Therefore, the *in vitro* findings should be viewed as complementary evidence supporting the *in vivo* observations.

Mechanistically, copper primarily enters cells *via* CTR1 and participates in the regulation of mitochondrial energy metabolism and redox homeostasis. Aberrant enhancement of CTR1 expression or activity can lead to dysregulated copper influx, thereby disrupting cellular copper homeostasis and creating conditions conducive to the induction of cuproptosis (Öhrvik and Thiele, 2014). Consistent with this notion, our transcriptomic analyses and *in vivo* and *in vitro* validations show that CTR1 expression is markedly upregulated after ICH, accompanied by characteristic alterations in cuproptosis-related molecular signatures. Among these, FDX1 and LIAS serve as key nodes within the mitochondrial lipoylation pathway, jointly maintaining the homeostatic coupling between protein lipoylation and mitochondrial metabolic function (Dreishpoon et al., 2023; Lu et al., 2025). In contrast, GLS, an important negative regulator of cuproptosis, is significantly downregulated after ICH and partially restored following intervention, consistent with its protective role (Zhang et al., 2024). Classical cuproptosis models generally regard the abnormal aggregation of lipoylated proteins as a core molecular event of cuproptosis and consider FDX1 to be closely associated with mitochondrial protein lipoylation levels (Li et al., 2025). However, disease-related models suggest that this relationship is not strictly linear or invariant. Under conditions of pathological copper overload accompanied by sustained metabolic stress, lipoylation may be reduced despite FDX1 upregulation, potentially reflecting altered stability of lipoylated proteins or impaired lipoylation capacity, manifesting as decreased DLAT lipoylation together with enhanced oligomerization (Liu et al., 2025; Guo et al., 2026). Accordingly, the increase in FDX1 together with reduced mitochondrial lipoylation signals observed in the ICH context may reflect disruption of lipoylated protein homeostasis under pathological copper accumulation, rather than a simple linear relationship between FDX1 expression and DLAT lipoylation. Moreover, the enhancement of protein oligomerization further indicates abnormal aggregation of lipoylated substrates, which is consistent with a key molecular feature of cuproptosis. Nevertheless, the present data cannot determine whether the reduction in DLAT lipoylation is mainly due to enhanced degradation, impaired lipoylation machinery, or redistribution into oligomeric forms. Further studies using protein half-life analysis, copper chelation, or related approaches will be required to clarify this mechanism (Karbowski et al., 2022).

At the level of upstream regulation, our findings suggest that Sp1 may act as an important transcriptional regulator linking the ICH-induced stress environment to remodeling of copper homeostasis. Both *in vivo* and *in vitro* experiments consistently showed that Sp1 expression is elevated after ICH and was accompanied by increased nuclear localization and promoter binding activity, together with CTR1 upregulation and intracellular copper accumulation. Previous studies have demonstrated that Sp1 can be activated under various oxidative and metabolic stress conditions and regulates the transcription of genes involved in stress responses and metabolic regulation through binding to GC-rich promoter elements (Kaczynski et al., 2003; Ryu et al., 2003; O’Connor et al., 2016). In the context of copper homeostasis, Sp1 is recognized as a key transcription factor for CTR1, and alterations in its activity can directly influence cellular copper uptake (Song et al., 2008). Building on this, ChIP–qPCR showed that Sp1 binding to the CTR1 promoter was significantly enhanced under ICH pathological conditions, providing molecular evidence supporting involvement of the Sp1/CTR1 axis in this context. Nevertheless, Sp1 activation should not be inferred from total Sp1 expression alone. In the present study, evidence for Sp1/CTR1-axis activation was based on combined changes in Sp1 expression, nuclear localization, promoter binding, and downstream CTR1 expression. However, Sp1 transcriptional activity may also be modulated by post-translational regulation, redox status, and other metal-homeostasis changes under ICH-related stress conditions. In addition, because MA is not entirely specific for Sp1, these findings do not exclude possible contributions from other Sp-family proteins or parallel transcriptional pathways affected by MA. Future studies using Sp1-or CTR1-specific genetic manipulation and more direct assessments of Sp1 activity will be required to further define the causal contribution and specificity of this pathway. Notably, in contrast to studies that primarily emphasize changes in copper concentration *per se* (Liu et al., 2024), our results suggest that under the combined oxidative and metabolic stress characteristic of ICH, copper homeostasis disruption is not merely a passive consequence but is more likely subject to active amplification at the transcriptional level. Distinct from previous investigations that largely examined Sp1/CTR1 regulation under homeostatic or non-neuronal conditions (Song et al., 2008; Liang et al., 2012), the present study suggests aberrant activation of this axis at the transcriptional level in the ICH pathological setting and supports its association with aggravated copper dyshomeostasis. Furthermore, pharmacological inhibition of Sp1 with MA concurrently attenuated CTR1 upregulation, intracellular copper accumulation, and cuproptosis-related metabolic abnormalities, accompanied by improvement in neuronal structural damage. These findings are consistent with prior reports that inhibition of copper uptake alleviates copper-dependent cellular injury (Barca et al., 2019), while extending this concept by shifting the regulatory level upstream to the transcription factor, thereby suggesting that Sp1 may participate in secondary metabolic injury cascades through activation of the copper uptake pathway (Fitisemanu and Padilla-Benavides, 2024).

As cuproptosis-related molecular events progress, they may be accompanied by compromised neuronal axonal structural integrity and impaired reparative capacity. Maintenance of axonal structure and function is highly dependent on mitochondrial energy supply and the continuity of axonal transport, both of which are particularly vulnerable to transport blockade and structural disruption during the exacerbation of secondary injury after ICH (Wang et al., 2021; Pasokh et al., 2025). In this study, we observed increased APP expression and decreased GAP43 expression at 72 h, consistent with morphological features of axonal fragmentation and beading, indicating that this stage is characterized by a predominantly “injury-dominant,” rather than “regeneration-dominant,” pathological state. Aberrant accumulation of APP within axons is widely regarded as a sensitive indicator of impaired axonal transport and structural damage, in agreement with our observations (Johnson et al., 2013; Xiong et al., 2023). In contrast, changes in GAP43 expression exhibit pronounced temporal specificity, reflecting whether axonal growth and regenerative programs are effectively initiated rather than serving as an immediate marker of injury. Previous studies reporting increased GAP43 expression have predominantly described later or repair-associated stages or contexts involving repair-promoting interventions, where such increases are typically interpreted as compensatory axonal regeneration or enhanced plasticity (Cui et al., 2017), and do not imply uniform upregulation across all stages after ICH. The 72 h time point examined in the present study more closely corresponds to a phase of markedly aggravated secondary injury, during which brain edema approaches its peak and is accompanied by substantial neurological deficits (Liu et al., 2022). At this stage, mitochondrial metabolic dysfunction and oxidative stress predominate, such that axonal repair programs have not yet been effectively initiated and may even be suppressed, resulting in downregulation of GAP43. Consistently, prior ICH-related studies have also reported decreases in GAP43 during the acute or early phase, followed by gradual recovery with disease progression or reparative interventions (Guo et al., 2018; Sandelius et al., 2018; Chen et al., 2021). Therefore, the reduction in GAP43 observed at 72 h in this study is not inconsistent with existing literature, but rather reflects a pathological state in which injury predominates over regeneration within this specific temporal window. Nevertheless, GAP43 is an axonal growth–associated marker rather than a direct functional measure of axonal regeneration. Therefore, the present findings should be interpreted as changes in axonal injury- and growth-associated molecular markers, and further functional approaches, such as electrophysiological assessment or axonal tract tracing, will be required to directly evaluate axonal regeneration and circuit recovery.

Several limitations of the present study should be acknowledged. Pharmacological inhibition of Sp1 with MA may affect multiple transcriptional pathways, because MA is not entirely specific for Sp1 and may also interfere with other Sp-family transcription factors. Therefore, the protective effects observed after MA treatment cannot be exclusively attributed to Sp1 inhibition or to regulation of the Sp1/CTR1 axis. In addition, although 100 nM MA did not markedly reduce cell viability under normal conditions in the CCK-8 assay, MA-only controls were not systematically included in the subsequent biochemical analyses. Although our results support the involvement of Sp1/CTR1-associated regulation in ICH-related copper dyshomeostasis and neuronal injury, more specific genetic validation, particularly through the use of neuron-specific Sp1 conditional knockout mice and targeted CTR1 knockdown/knockout, is still needed to further clarify the causal role and specificity of this pathway. The small sample size used in some *in vivo* molecular analyses may reduce the stability of variance estimates and increase sensitivity to individual observations. Finally, assessment of axonal injury in the present study was mainly based on molecular markers and morphological observations, and more direct functional validation is still required.

In summary, pathological stress following ICH was associated with increased Sp1 activity and CTR1 expression, together with neuronal copper overload, cuproptosis-associated molecular alterations, and axonal injury-related molecular and morphological changes. Pharmacological inhibition of Sp1 was associated with attenuation of these changes (Figure 7). Collectively, these findings support a potential involvement of the Sp1/CTR1 axis in secondary neuronal injury after ICH and suggest that this pathway may warrant further investigation in the context of therapeutic development.

**FIGURE 7:**
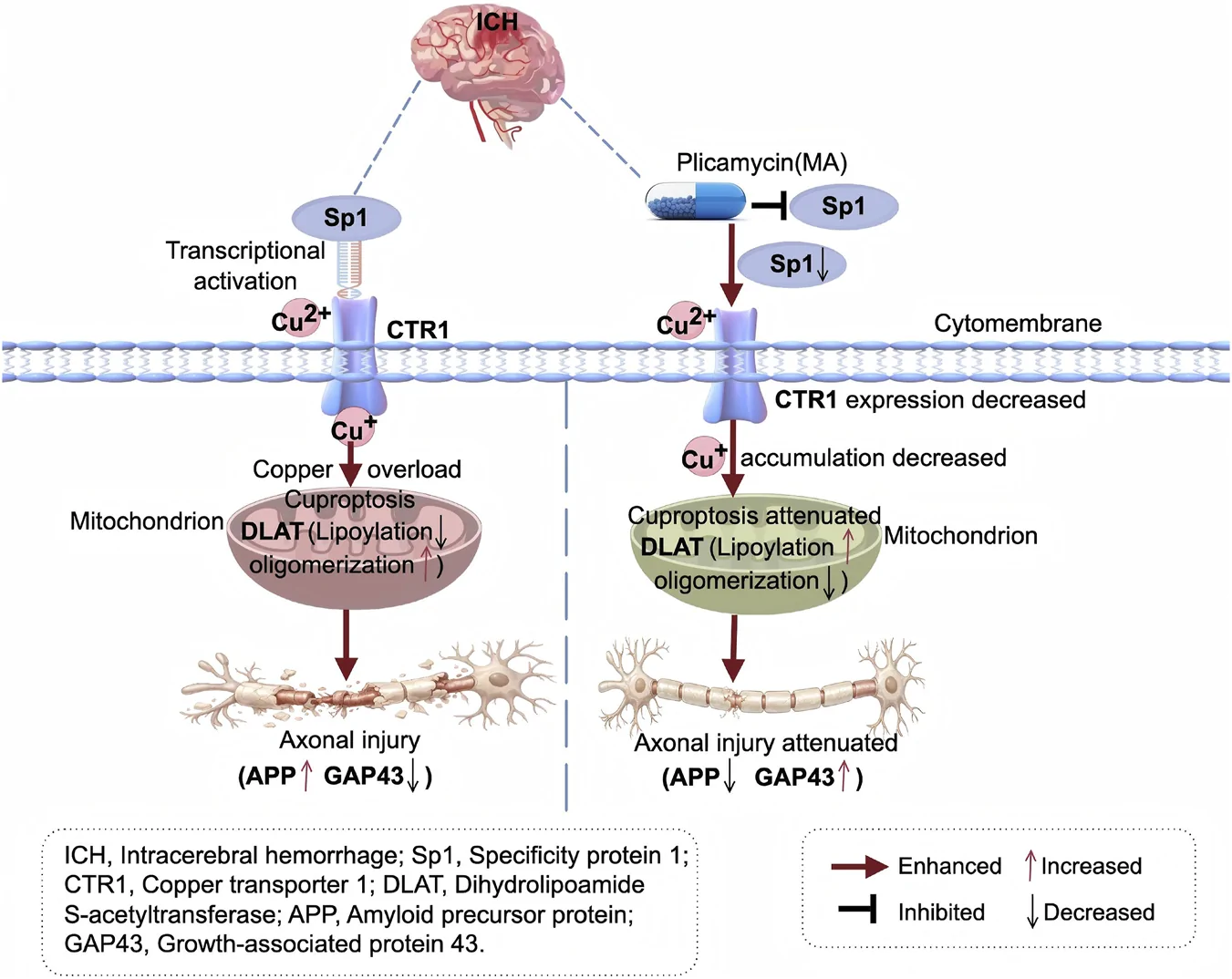
Proposed model showing how inhibition of the Sp1/CTR1 axis may attenuate cuproptosis-associated molecular alterations and axonal injury-related changes after ICH After ICH, activation of Sp1 may enhance CTR1 transcription, leading to increased neuronal copper influx and disruption of copper homeostasis, which may contribute to cuproptosis-associated molecular alterations and axonal injury-related molecular and morphological changes. Inhibition of the Sp1/CTR1 axis attenuates these changes.

## Data Availability

The RNA-sequencing dataset generated in this study has been deposited in the NCBI Gene Expression Omnibus under accession number GSE326178.

## Supplementary material

SUPPLEMENTARY FIGURE S1 Western blot analysis of cuproptosis-related protein changes at the cellular level. Western blot analysis and densitometric quantification of the cuproptosis-related proteins CTR1, FDX1, LIAS, GLS, and DLAT lipoylation at different time points in the cell model.

SUPPLEMENTARY FIGURE S2 Immunofluorescence analysis of cuproptosis-associated metabolic factors in the cell model. Representative immunofluorescence images and quantitative analysis of FDX1, LIAS, and GLS in the cell model. Nuclei were counterstained with DAPI. Scale bar = 20 μm.

## Glossary

APP: amyloid precursor protein
CCK-8: Cell Counting Kit-8
ChIP: chromatin immunoprecipitation
ChIP–qPCR: chromatin immunoprecipitation coupled with qPCR
Ctrl: control
CTR1(Slc31a1): copper transporter 1
Cu/Zn-SOD: copper–zinc superoxide dismutase
DEGs: differentially expressed genes
DLAT: dihydrolipoamide S-acetyltransferase
FDX1: ferredoxin 1
GO: Gene Ontology
GLS: glutaminase
GAPDH: glyceraldehyde-3-phosphate dehydrogenase
GAP43: growth-associated protein 43
ICP-MS: inductively coupled plasma mass spectrometry
ICH: intracerebral hemorrhage
KEGG: Kyoto Encyclopedia of Genes and Genomes
LIAS: lipoic acid synthetase
MA: Mithramycin A (Plicamycin)
NeuN: neuronal nuclei
PI3K–Akt: phosphoinositide 3-kinase–Akt pathway
PCR: polymerase chain reaction
PPI: protein–protein interaction
qPCR: quantitative polymerase chain reaction
RN-C: Rat cortical neuron–related cells
RT-qPCR: real-time quantitative polymerase chain reaction
RNA-seq: RNA sequencing
SPF: specific pathogen-free
Sp1: specificity protein 1
SD: Sprague–Dawley
TCA: tricarboxylic acid
p53: tumor protein p53
VDAC1: voltage-dependent anion channel 1
