# Supplementary material for: Inhibition of the Sp1/CTR1 axis attenuates cuproptosis and alleviates axonal injury following intracerebral hemorrhage
Source: Front Mol Biosci. 2026 Sep 14;13:1932767. doi: 10.3389/fmolb.2026.1932767 (PMC13616648; doi:10.3389/fmolb.2026.1932767)
Supplement: Supplementary file 1 [file Table1.docx]

**Supplementary Table S1. Antibodies used in this study**

| **Application** | **Antibody** | **Host species** | **Supplier** | **Catalog No.** | **Dilution / Amount** |
| --- | --- | --- | --- | --- | --- |
| WB | Sp1 | Rabbit | Proteintech | 21962-1-AP | 1:12000 |
| WB | LIAS | Rabbit | Proteintech | 11577-1-AP | 1:4000 |
| WB | FDX1 | Rabbit | Proteintech | 12592-1-AP | 1:3000 |
| WB | GLS | Rabbit | Proteintech | 12855-1-AP | 1:42000 |
| WB | DLAT | Rabbit | Proteintech | 13426-1-AP | 1:8000 |
| WB | APP | Mouse | Proteintech | 60342-1-Ig | 1:3000 |
| WB | GAP43 | Rabbit | Proteintech | 16971-1-AP | 1:8000 |
| WB | CTR1 | Rabbit | Bioswamp | PAB36840 | 1:1200 |
| WB | Lipoic Acid | Mouse | Santa Cruz Biotechnology | sc-101354 | 1:4000 |
| WB | GAPDH-HRP | Not specified | Servicebio | ZB15004-HRP | 1:3000 |
| WB secondary | HRP-conjugated goat anti-rabbit IgG (H+L) | Goat | Proteintech | RGAR001 | 1:10000 |
| WB secondary | HRP-conjugated goat anti-mouse IgG (H+L) | Goat | APExBIO | K1221 | 1:10000 |
| IF | Sp1 | Mouse | Proteintech | 66508-1-Ig | 1:1000 |
| IF | APP | Mouse | Proteintech | 60342-1-Ig | 1:150 |
| IF | VDAC1 | Rabbit | Proteintech | 10866-1-AP | 1:300 |
| IF | Lipoic Acid | Mouse | Santa Cruz Biotechnology | sc-101354 | 1:1000 |
| IF | CTR1 | Rabbit | Bioss | bs-10773R | 1:300 |
| IF | LIAS | Rabbit | Proteintech | 11577-1-AP | 1:200 |
| IF | FDX1 | Rabbit | Proteintech | 12592-1-AP | 1:400 |
| IF | GLS | Rabbit | Proteintech | 12855-1-AP | 1:200 |
| IF | NeuN-BF647 conjugated | Rabbit | Bioss | bs-10394R-BF647 | 1:200 |
| IF secondary | HyperFluor™ 594 Goat Anti-Mouse IgG (H+L) Antibody | Goat | APExBIO | K3306 | 1:500 |
| IF secondary | HyperFluor™ 488 Goat Anti-Rabbit IgG (H+L) Antibody | Goat | APExBIO | K1206 | 1:500 |
| ChIP-qPCR | Sp1 | Rabbit | Thermo Fisher Scientific | MA5-35331 | 5 μg/reaction |
| ChIP-qPCR | Histone H3 | Rabbit | Abcam | ab1791 | 5 μg/reaction |
| ChIP-qPCR | IgG | Rabbit | Abcam | ab172730 | 5 μg/reaction |
